# Editorial for the Special Issue on Recent Advances in Inkjet Technology

**DOI:** 10.3390/mi14030681

**Published:** 2023-03-19

**Authors:** Dong Kee Sohn

**Affiliations:** School of Mechanical Engineering, Sungkyunkwan University, Suwon 16419, Republic of Korea; dksohn@skku.edu

Inkjet is a well-established technology that has been applied in various applications ranging from graphical printing to functional material printing. As an additive process in manufacturing technology, inkjet has many advantages over conventional subtractive manufacturing technology [1,2]. It is advantageous as it is a simple process, facilitates a reduction in waste materials, is scalable, and is less dependent on target morphology. Evidently, it also has various shortcoming, including the long process time, a bulky ink supply system, limited material loading, and post-treatment complexities. Inkjet technology can be divided into three actuating sources, consisting of continuous, thermal, and piezoelectric categories. The electro-hydrodynamic jet is also an alternative to those driving sources, but it is in the early stages of commercialization [3]. Piezo inkjet technology stands out as a promising manufacturing method, thanks to its versatility in accommodating a diverse range of ink materials. A severe worldwide economic crisis in 2007 had a profound impact on the home and office inkjet market and accelerated the expansion of inkjet technology applications in various fields. The graphic printing market is very large and covers everything from home and office document printing to textile and package printing. Inkjet technology also covers additive manufacturing technology.

Additive manufacturing fields using inkjet printing have made several advances, including printed electronics and biomaterial printing. The PCB manufacturing process, including passive components, was among the first in printed electronics to adopt inkjet printing [4], and, since then, significant efforts have been made to incorporate inkjet printing into the production of active components [5,6]. OLED displays have shown promising results and are on the brink of becoming a commercially available products. Most notably, QD display relies more on the inkjet process due to its low temperature and atmospheric pressure processing characteristics. In bio-material printing, inkjet is utilized as a dispensing method with a higher resolution [7]. Inkjet is not different from the squeezer-type dispenser and its very small dispensing ability enables its own application to gene chips, drug release and screening, tissue repair, etc.

Inkjet is a collection of technologies in head design, fabrication, operation, ink material selection, manufacturing, and application [8]. Since it was first developed some time ago, extensive foundational research in this area has already been completed. However, due to the basic complexity of inkjet printing and the demand for higher resolution, throughput, and material loading, various studies on the evolution of inkjet technology for defect-free printing are still in progress. As piezoelectric inkjet heads employ more nozzles with smaller diameters, inkjet drivers have changed from older bulk piezo actuators to thin-film piezo actuators. As the drop size decreased, the pulse width reduced, and the driving waveform modulation became very difficult. Accordingly, it has become very important to enable stable drop discharge, even with a simple drive waveform. Consideration should be given to minimizing clogging, which is made easier by smaller nozzle sizes. Efforts to reduce discharge interference between nozzles may be required for the narrowed nozzle pitch. On the ink side, efforts have been made to increase the loading of functional materials, enhance the jetting stability, and obtain good surface morphology [9,10,11]. In addition, UV-curable inks or inks in various solvents suitable for target surface and post-processing applications have been developed.

Significant progress has been made in the field of inspection as well. In the field of inspecting the surface of objects manufactured with inkjet, technology has advanced and accessibility to equipment has improved. However, in terms of drop observation, the basic configuration has not changed much over the years. As various applications require in situ drop monitoring, it will be necessary to develop this.

This Special Issue covers eight recent research papers related to inkjet printing. While the number of studies is limited, they demonstrate a diverse array of results. Al-Halhouli et al. [12] reviewed bio and wearable sensors manufactured by the printing process. With the monitoring of common symptoms related to infectious diseases by printed wearable sensors, epidemic disease can be controlled effectively. Sieber et al. [13] proposed a process flow for high-resolution 3D manufacturing by inkjet printing. This work is expected to play an important role as it adopt state-of-the-art digital twin for the commercialization of inkjet-printed products in the future. Graf et al. [14] presented a complete work process of manufacturing polymer–ceramic composites by 3D inkjet printing. This article covered everything from ink formulation to product testing, showing how real inkjet printing products are developed. Karaş et al. [15] made a strain gauge sensor on a carbon-fiber-reinforced polymer surface via direct inkjet printing. Their research focused on the excellent adhesion of inkjet-printed patterns and obtained good results. Kim et al. [16] developed a computational model for simulating inkjet ejection, including pressure wave propagation and actuator motion. In addition, the ink supply effect on the inkjet performance and meniscus movement, which is rarely covered, was also studied. Chen et al. [17] made an antenna on a wound dressing with inkjet printing. The findings of this study demonstrated how to obtain a silver film with the necessary qualities. Cavaleiro de Ferreira et al. [18] developed a very simple and low-cost drop monitoring system. The team attempted to utilize readily available components, resulting in a system that can be easily replicated by anyone. Hussain et al. [19] focused on a non-Newtonian polymer ink. Due to the very small time scale of the physics of the flow in inkjet head, conventional representation of the complex viscosity and shear modulus need to be analyzed to higher frequency and this work presented inkjet stability based on these characteristics.

When implementing necessary functions through inkjet printing, it is important to demonstrate excellence compared to other manufacturing processes by making full use of the advantages of inkjet. Through persistent research and development, inkjet technology is expected to be a viable additive manufacturing process.
